# A PAS Protein Directs Metabolic Reprogramming during Cryptococcal Adaptation to Hypoxia

**DOI:** 10.1128/mBio.03602-20

**Published:** 2021-03-16

**Authors:** Youbao Zhao, Xiaorong Lin

**Affiliations:** a College of Veterinary Medicine, Henan Agricultural University, Zhengzhou, Henan Province, People’s Republic of China; b Department of Microbiology, University of Georgia, Athens, Georgia, USA; Duke University Medical Center

**Keywords:** Snf1, Sre1, carbon metabolism, ergosterol, hypoxia, metabolism, obligate aerobe, transcription factors

## Abstract

To aerobic organisms, low oxygen tension (hypoxia) presents a physiological challenge. To cope with such a challenge, metabolic pathways such as those used in energy production have to be adjusted. Many of such metabolic changes are orchestrated by the conserved hypoxia-inducible factors (HIFs) in higher eukaryotes. However, there are no HIF homologs in fungi or protists, and not much is known about conductors that direct hypoxic adaptation in lower eukaryotes. Here, we discovered that the transcription factor Pas2 controls the transcript levels of metabolic genes and consequently rewires metabolism for hypoxia adaptation in the human fungal pathogen Cryptococcus neoformans. Through genetic, proteomic, and biochemical analyses, we demonstrated that Pas2 directly interacts with another transcription factor, Rds2, in regulating cryptococcal hypoxic adaptation. The Pas2/Rds2 complex represents the key transcription regulator of metabolic flexibility. Its regulation of metabolism rewiring between respiration and fermentation is critical to our understanding of the cryptococcal response to low levels of oxygen.

## Abstract (precis)

C. neoformans is the main causative agent of fungal meningitis that is responsible for about 15% of all HIV-related deaths. Although an obligate aerobic fungus, C. neoformans is well adapted to hypoxia conditions that the fungus could encounter in the host or the environment.

## INTRODUCTION

Oxygen, as the electron acceptor in the generation of ATP via aerobic respiration, is required for many essential biochemical reactions. Deprivation of oxygen (hypoxia) presents a physiological and pathophysiological challenge to aerobic organisms, humans and microbes alike. In mammals, cells adjust to hypoxia by rewiring the route of ATP production and redox balance, which is achieved mainly through regulation by hypoxia-inducible factors (HIFs) (1). Under hypoxia, HIFs mediate the switch of ATP production from oxidative phosphorylation (OXPHOS) to glycolysis by upregulating a series of genes that lead to active glycolytic metabolism and inhibition of mitochondrial function (1). For instance, HIFs activate the transcription of genes encoding glycolytic enzymes (2, 3) and pyruvate dehydrogenase kinase (PDK) (4, 5), which in turn deactivates pyruvate dehydrogenase (PDH), the enzyme responsible for the conversion of pyruvate to acetyl coenzyme A (acetyl-CoA) that feeds the tricarboxylic acid (TCA) cycle (4). In addition, HIFs promote pyruvate reduction to lactate by activating lactate dehydrogenase A (LDHA) (6). These changes shunt pyruvate away from mitochondrial oxidation and regenerate NAD^+^ to permit continued glycolysis and, consequently, continued ATP production under hypoxia. Thus, under hypoxia, HIFs increase glucose/carbon flux through glycolysis while minimizing input into the TCA cycle and oxidative phosphorylation.

The main features of the hypoxia response in fungal species tested resemble those observed in mammals. In the facultative aerobic yeast Candida albicans, hypoxia leads to the induction of genes in glycolysis and the repression of those in aerobic respiration (7–9). A metabolomics analysis of *Candida* cells encountering hypoxia further confirmed such metabolic reprogramming (10). In the obligate aerobic mold Aspergillus nidulans, exposure to hypoxia results in an increase in the transcript levels of genes involved in glycolysis, fermentation, and the γ-aminobutyrate (GABA) shunt, which bypasses two steps of the tricarboxylic acid (TCA) cycle (11). Similarly, transcriptomic and proteomic studies in a related species, Aspergillus fumigatus, under hypoxia revealed increases in the oxidative stress response, glycolysis and fermentation, cell wall biosynthesis, and iron metabolism with concomitant decreases in the TCA cycle (12). Although Cryptococcus neoformans is an obligate aerobic basidiomycete and is evolutionarily distant from the above-mentioned ascomycetes, microarray-based transcriptional profiling showed the upregulation of genes involved in stress and carbohydrate uptake/metabolism in response to hypoxic conditions (13, 14), similar to what has previously been reported in Saccharomyces cerevisiae (15), Schizosaccharomyces pombe (16), and C. albicans (8). Thus, like mammals, fungi adjust to hypoxia through reprogramming metabolism to generate ATP and balance redox potential. To our knowledge, no homologs of HIFs have been identified in lower eukaryotes that regulate the metabolic response to hypoxia.

In fungi, sterol regulatory element (SRE) binding protein (SREBP) transcription factors are known to be involved in hypoxic adaptation when the sterol level in the membrane drops in response to limited oxygen (17). SREBPs are endoplasmic reticulum (ER) membrane-bound transcription factors. The N terminus of SREBP is a basic helix-loop-helix (bHLH) leucine zipper, and the C terminus forms a tight complex with SREBP cleavage-activating protein (SCAP), which functions as a sensor for ergosterol levels. In sterol-depleted cells, SREBP-SCAP is sorted from the ER to the Golgi complex, where the N-terminal transcription factor domain of SREBP is liberated from the membrane by proteolytic cleavage. The released N-terminal segment of SREBP translocates into the nucleus, where it binds to SREs present in the promoters and executes its transcriptional regulatory function. The fungal SREBP pathway appears to be diverse among different species. For instance, SREBP and SCAP homologs are present in S. pombe and C. neoformans but absent in S. cerevisiae and C. albicans, while A. fumigatus contains the SREBP but not the SCAP homolog. In A. fumigatus, the SREBP SrbA regulates ergosterol biosynthesis, iron uptake, nitrate assimilation, and heme biosynthesis in response to hypoxia (18). The other potential SREBP, SrbB, coregulates heme biosynthesis together with SrbA. SrbB may also regulate carbohydrate and lipid metabolism under hypoxia independent of SrbA (18). In C. neoformans, the SREBP Sre1 is required for the induction or repression of only a small portion of hypoxia-responsive genes, most of which are involved in ergosterol biosynthesis or have no annotated functions (13, 14). The published data support that SREBPs are largely dispensable for the induction of glycolytic genes and the suppression of mitochondrial OXPHOS genes in response to hypoxia in fungi (12–14, 16, 19).

Glycolysis is a central metabolic pathway that assimilates carbohydrates for either respiration or fermentation. Carbohydrates entering glycolysis are converted to the key metabolite pyruvate while producing ATP and NADH. Pyruvate, depending on the availability of oxygen, is used for energy production through respiration or fermentation. Despite the central and conserved role of glycolysis, its regulation might be distinct among different fungal species. In the facultative anaerobe S. cerevisiae, the transcription regulators Gcr1 and Gcr2 are primarily responsible for the activation of the expression of the glycolytic genes when cells are cultured on fermentable carbon sources, while they are dispensable for growth on nonfermentable carbon sources (20, 21). However, most organisms do not have Gcr1 or Gcr2 homologs (22). In C. albicans, Tye7 and Gal4 are responsible for the induction of the glycolytic genes under hypoxic conditions, and their genetic inactivation leads to a substantial growth defect under hypoxia (7). In A. fumigatus, the carbon repression regulator CreA promotes fungal fitness in low-oxygen infection microenvironments (23). Mig1 and Fzc36 are the cryptococcal homologs of CreA and Gal4 (24, 25). However, they do not appear to be involved in hypoxia adaption based on a preliminary screen of the gene deletion mutants in the publicly available deletion sets. There is no predicted Tye7 homolog in the genome of C. neoformans.

In this study, we sought to investigate the regulation of metabolic reprogramming in response to hypoxia in C. neoformans, an obligate aerobic basidiomycete that is distantly related to the ascomycetes discussed above. Given the technical limitations of previous microarray studies, we conducted RNA deep sequencing experiments to gain a more holistic transcriptomic response of C. neoformans to hypoxia. We observed dramatic changes in metabolism-related genes, including the induction of glycolysis and the repression of OXPHOS, corroborating the importance of metabolic rewiring in hypoxia adaptation in C. neoformans. More importantly, our genetic and biochemical studies led us to the discovery that the transcription factor complex Pas2/Rds2 controls metabolic reprogramming in cryptococcal hypoxia adaptation. Homologs of the Pas2/Rds2 complex exist in ascomycetous fungi such as S. cerevisiae (26, 27) and A. nidulans (28, 29), although their role in hypoxia adaptation has not been tested. Given that basidiomycetes such as *Cryptococcus* diverged from ascomycetes from a common ancestor at least 600 million years ago (30), the Pas2/Rds2 complex and its regulatory pathways might be conserved among diverse fungal species. Future investigation into the relationship between the Pas2/Rds2 complex and other regulators identified in different species could reveal the conserved and distinct features of metabolism rewiring in response to hypoxia.

## RESULTS

### Transcriptomic changes indicate metabolic reprogramming in C. neoformans in response to hypoxia.

As an obligate aerobe, C. neoformans adapts remarkably well to conditions with an extremely low level of oxygen. To gain a holistic understanding of transcriptomic changes in C. neoformans in response to such extreme hypoxic conditions, we conducted a comparative transcriptome analysis by RNA sequencing (RNA-seq) under normoxia and hypoxia. We first cultivated the fungus on solid yeast extract-peptone-dextrose (YPD) medium under normoxia overnight and then transferred the plates to a hypoxia chamber with the oxygen level controlled at 0.1% for 3 h. Cells that continued to be cultured under normoxia (21% partial O_2_ pressure [pO_2_]) served as controls. *Cryptococcus* cells were then harvested for RNA extraction and subsequent sequencing.

**FIG 1:**
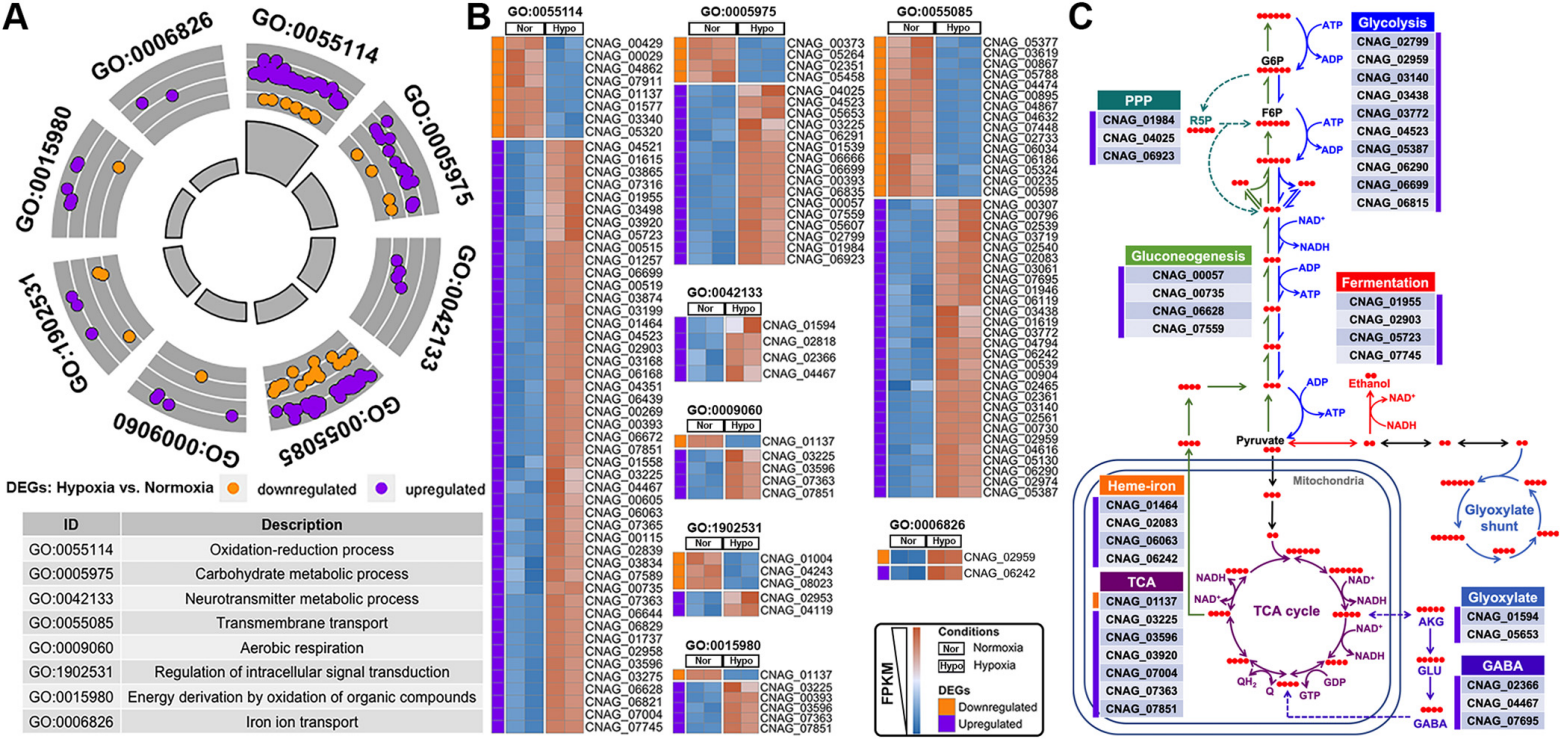
C. neoformans cells reprogram central carbon metabolism in response to hypoxia. (A) GOCircle of significantly changed functional categories of DEGs of wild-type cells under hypoxia relative to normoxia conditions. The dots indicate the upregulated and downregulated genes within a specific GO category. The gray bars in the center indicate the *P* values of GO terms, with higher bars representing higher significance of the GO term category. The description of GO categories is listed in the table at the bottom. (B) Heat map of the DEGs within the significantly changed GO categories under hypoxia relative to normoxia conditions. FPKM, fragments per kilobase per million. (C) DEGs that are involved in central metabolism pathways in response to hypoxia. See the details about the DEGs in Data Set S1 to Data Set S3 in the supplemental material. Red dots represent the number of carbon atoms. G6P, glucose-6-phosphate; F6P, fructose-6-phosphate. AKG, alpha-ketoglutarate; GLU, glutamic acid.

We considered genes showing at least 2-fold changes in transcript levels to be differentially expressed (adjusted *P* value of <0.05; log_2_ fold change [log_2_FC] greater than 1 or less than −1). In total, 356 genes were upregulated and 338 genes were downregulated in C. neoformans during growth under hypoxia relative to normoxia conditions (see Data Set S1 in the supplemental material). As expected, the transcript levels of some mitochondrial genes involved in OXPHOS were significantly downregulated due to the restricted level of oxygen, the final electron acceptor (Data Set S1). To gain a general overview of the biological processes affected by hypoxia, we performed gene set enrichment analysis by pooling all the differentially expressed genes (DEGs) based on Gene Ontology (GO) classification. The significant categories under hypoxia include processes associated with oxidation-reduction, carbohydrate metabolism, neurotransmitter metabolism, transmembrane transport, aerobic respiration, intracellular signal transduction, energy derivation by the oxidation of organic compounds, and iron ion transport (Fig. 1A). Surprisingly, even though the numbers of upregulated and downregulated genes in response to hypoxia are comparable, most of the genes from these functional categories are upregulated in response to hypoxia, indicating that these genes play a positive role in regulating hypoxia adaptation (Fig. 1B and Data Set S2). We noticed that most of the significantly changed categories are related to metabolism (Fig. 1A). Further metabolic pathway enrichment analysis highlighted the central carbon metabolism pathways, including glycolysis, the pentose phosphate pathway (PPP), gluconeogenesis, and the TCA cycle (Fig. 1C and Data Set S3). Moreover, genes involved in fermentation, the glyoxylate shunt, and the GABA biosynthesis pathway, which contribute to redox balance, were also upregulated under hypoxia (Fig. 1C and Data Set S3). The upregulation of the iron transporter-encoding genes *CNAG_02083* (*SIT2*) (3.8-fold) and *CNAG_06242* (*CFT1*) (3.6-fold) implicates an increased need for iron under hypoxia, in agreement with previously reported findings from *Cryptococcus* (13, 14) and other fungi (7, 8, 12, 31). The heme biosynthesis-related gene *CNAG_06063* (*COX15*) (2.0-fold) together with the heme binding flavohemoglobin-encoding gene *CNAG_01464* (*FHB1*) (4-fold) were also upregulated under hypoxia (Fig. 1C and Data Set S3). The transcriptome data suggest that in response to hypoxia, *Cryptococcus* reshuffles metabolic pathways to alter energy production from OXPHOS to substrate-level phosphorylation and to rebalance the redox status through upregulating fermentation, glyoxylate, and GABA shunts. Thus, consistent with what has been observed in other organisms, C. neoformans reprograms metabolism in adaptation to hypoxia.

10.1128/mBio.03602-20.5 DATA SET S1 List of DEGs in the wild type under hypoxia versus normoxia. Download Data Set S1, XLSX file, 0.06 MB. Copyright © 2021 Zhao and Lin. 2021 Zhao and Lin. https://creativecommons.org/licenses/by/4.0/ This content is distributed under the terms of the Creative Commons Attribution 4.0 International license.

10.1128/mBio.03602-20.6 DATA SET S2 GO categories of DEGs in the wild type under hypoxia versus normoxia. Download Data Set S2, XLSX file, 0.02 MB. Copyright © 2021 Zhao and Lin. 2021 Zhao and Lin. https://creativecommons.org/licenses/by/4.0/ This content is distributed under the terms of the Creative Commons Attribution 4.0 International license.

10.1128/mBio.03602-20.7 DATA SET S3 List of differentially expressed metabolic genes in response to hypoxia. Download Data Set S3, XLSX file, 0.01 MB. Copyright © 2021 Zhao and Lin. 2021 Zhao and Lin. https://creativecommons.org/licenses/by/4.0/ This content is distributed under the terms of the Creative Commons Attribution 4.0 International license.

### Pas2 regulates hypoxic growth in an SREBP-independent manner.

The SREBP pathway regulates hypoxic growth in fungi (17), including C. neoformans (13, 14). However, previous microarray studies showed that only a small portion of transcript-level changes in C. neoformans under hypoxic treatment were *SRE1* dependent, most of which are involved in ergosterol biosynthesis (13, 14, 19). Our genome-wide transcriptomic data by RNA-seq also indicate a wide range of metabolism changes in addition to ergosterol and iron homeostasis. These findings strongly suggest the existence of regulators in addition to the known SREBP pathway that mediate the metabolic response to hypoxia in C. neoformans.

**FIG 2:**
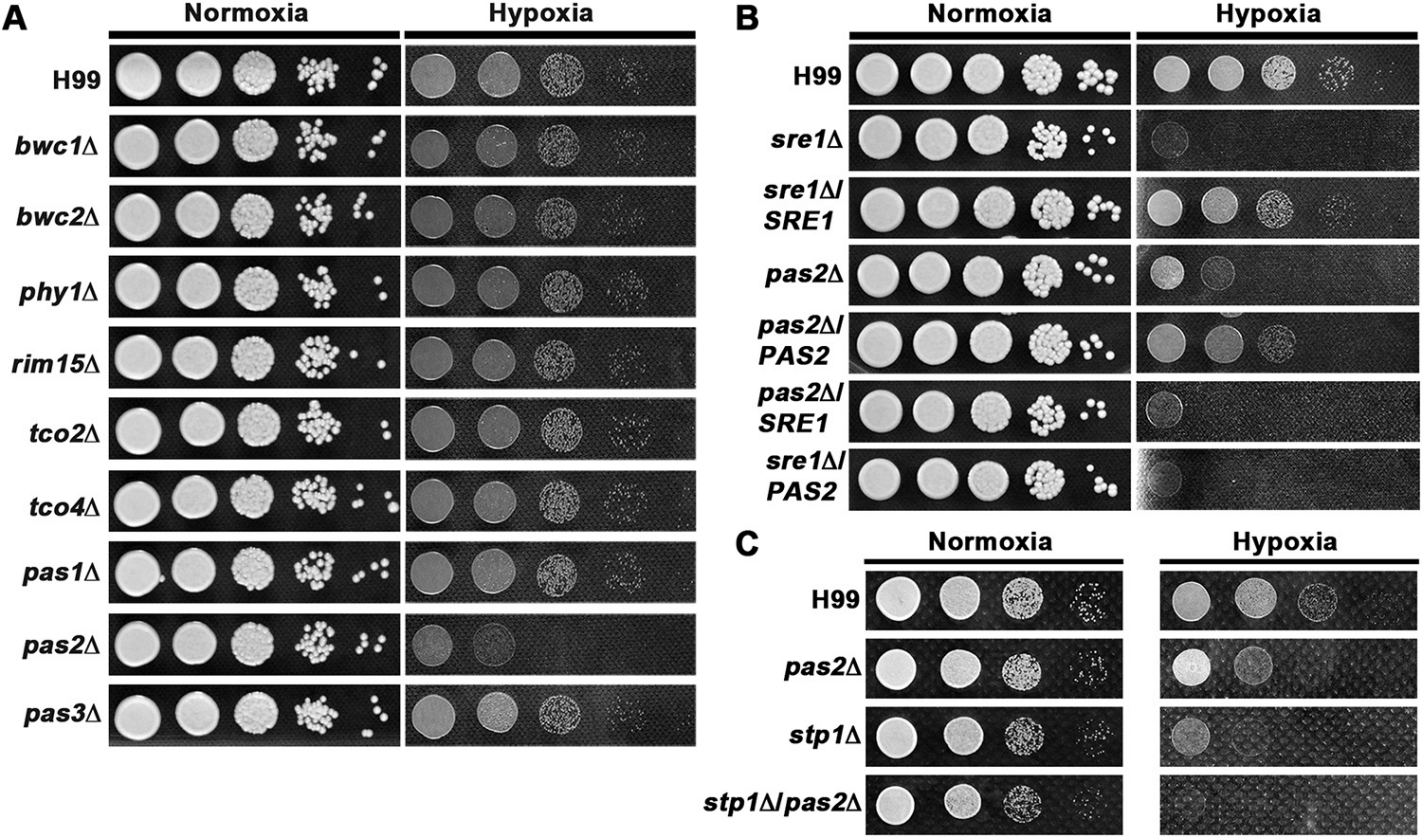
Pas2 regulates hypoxic growth in an SREBP-independent manner in C. neoformans. (A) Hypoxic growth of the nine PAS gene deletion mutants. Serial dilutions of cultures grown overnight were spotted onto YPD medium and cultured in a hypoxia chamber with 0.1% oxygen and 5% carbon dioxide at 37°C. Cells cultured in an incubator with 5% carbon dioxide at 37°C were set as the normoxia controls. Pictures were taken after 2 days of incubation with a fabric background. (B) Epistasis assay between *PAS2* and *SRE1* under hypoxia. (C) Epistasis assay between *PAS2* and *STP1* under hypoxia. Cells in panels B and C were cultured in the same way as described above for panel A.

In higher eukaryotes, metabolism reprogramming that leads to hypoxia adaptation is regulated by the PAS domain-containing heterodimers HIF1α and HIF1β (1). Orthologs of HIFs and key components of the HIF pathway are absent in lower eukaryotes (32, 33). We were also unable to identify any orthologs of HIFs in C. neoformans based on sequence similarity. Given that the PAS domains serve as internal sensors of oxygen and light across domains of life (34), we hypothesized that PAS domain-containing proteins might have regulatory functions in hypoxia adaptation in fungi. We previously found that C. neoformans carries nine PAS domain-containing genes in its genome (35). To investigate if any of the PAS proteins are involved in regulating cryptococcal hypoxic growth, we first examined the growth of the PAS gene deletion mutants in serotype A reference strain H99 using cobalt chloride as a hypoxia mimetic (19). Among the nine PAS knockout mutants, only the *pas2*Δ mutant grew poorly on cobalt chloride medium (Fig. S1A), indicating that Pas2 may regulate hypoxic growth in C. neoformans. To confirm the role of Pas2 in hypoxia growth, we cultured the PAS mutants in a hypoxic chamber. Consistently, only the *pas2*Δ mutant showed a significant growth defect under hypoxia (Fig. 2A). The deletion of *PAS2* in XL280, a serotype D reference strain, also resulted in a defect in growth under both cobalt chloride and hypoxia conditions (Fig. S1B). Furthermore, the ectopic introduction of a wild-type (WT) copy of the serotype A allele of *PAS2* into the *pas2*Δ mutant in either the serotype A or the serotype D background complemented the growth defect under both cobalt chloride and hypoxia conditions (Fig. S1B). As serotype A and serotype D diverged from a common ancestor 18.5 million years ago (36), the results indicate that the role of Pas2 in regulating hypoxic adaptation is conserved in the Cryptococcus neoformans species complex.

10.1128/mBio.03602-20.1 FIG S1 PAS2 regulates cell growth under CoCl_2_ and hypoxia conditions (Fig. 2). (A) Growth of PAS gene mutants on YES medium plus CoCl_2_. Cultures grown overnight were diluted to an OD_600_ of 3, serially diluted, spotted onto YES medium plus CoCl_2_, and cultured for 2 days. (B) Growth of PAS2-associated strains under CoCl_2_ and hypoxia conditions. Download FIG S1, TIF file, 2.6 MB. Copyright © 2021 Zhao and Lin. 2021 Zhao and Lin. https://creativecommons.org/licenses/by/4.0/ This content is distributed under the terms of the Creative Commons Attribution 4.0 International license.

As mentioned above, the SREBP pathway regulates cryptococcal hypoxic growth. Indeed, the deletion of *SRE1* nearly abolished hypoxic growth (Fig. 2B), consistent with previous studies (13, 14). To examine whether Pas2 functions in an SREBP-dependent manner, we decided to dissect the genetic relationship between *PAS2* and the SREBP pathway by epistasis analysis. We found that neither introducing an extra copy of *PAS2* in the *sre1*Δ mutant nor introducing an extra copy of *SRE1* in the *pas2*Δ mutant restored the deletion mutant’s growth defect in hypoxia, although the introduction of a copy of *PAS2* or *SRE1* rescued the growth defect of their cognate deletion mutants (Fig. 2B). As the *sre1*Δ mutant cannot grow under hypoxia, it would be challenging to discern the relationship between Pas2 and Sre1 in hypoxia growth by analyzing the phenotype of the *sre1*Δ *pas2*Δ double mutant. Thus, we decided to utilize *STP1* of the SREBP pathway. *STP1* encodes the proteinase that cleaves Sre1, which enables the translocation of Sre1’s regulatory N terminus to the nucleus in response to hypoxia (17). The deletion of *STP1* results in a hypoxic growth defect but not as severe as the deletion of *SRE1* (Fig. 2B and C) (37). This more modest defect in the hypoxia growth of the *stp1*Δ mutant is likely due to some redundant activities of other proteinases that could cleave Sre1 albeit at a lower efficiency. Nonetheless, *STP1* allows us to test the genetic relationship between *PAS2* and the SREBP pathway in cryptococcal adaptation to hypoxia as both the *stp1*Δ and the *pas2*Δ single mutants showed modest defects in hypoxia growth. To this end, we generated the *stp1*Δ *pas2*Δ double mutant. If *PAS2* and *STP1* function in the same SREBP pathway, we expect the double mutant to behave like the single mutants. However, the double mutant showed a much more severe defect in hypoxic growth than either the *stp1*Δ or the *pas2*Δ single mutant alone (Fig. 2C). This further supports that Pas2 and the SREBP pathway likely regulate cryptococcal hypoxic growth independently.

### Deletion of *PAS2* abolishes the induction of metabolic genes under hypoxia.

**FIG 3:**
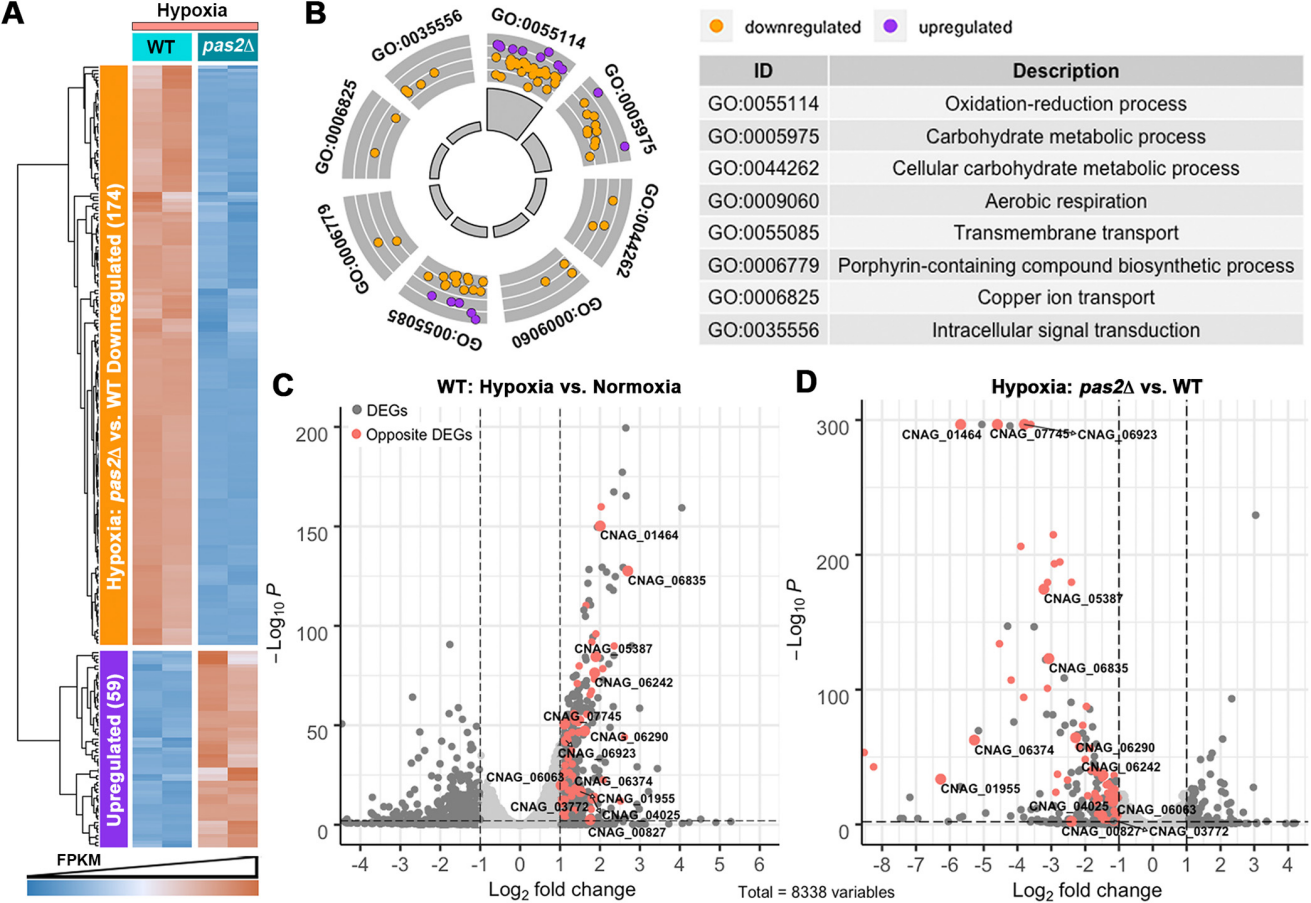
Deletion of *PAS2* abolishes the induction of metabolic genes in response to hypoxia in C. neoformans. (A) Heat map of the DEGs in comparison of the *pas2*Δ mutant and the WT under hypoxia. (B) GOCircle of significantly changed functional categories of DEGs in the *pas2*Δ mutant compared to the WT under hypoxia. The dots indicate the upregulated and downregulated genes within a specific GO category. The description of GO categories is listed in the table (Data Set S5). (C) Volcano plot of DEGs in the WT under hypoxia compared to normoxia. The colored dots indicate the DEGs that cannot be induced in the *pas2*Δ mutant under hypoxia. The gene identifications of DEGs that are involved in metabolism as shown in Fig. 1C are shown in the plot. (D) Volcano plot of DEGs in the *pas2*Δ mutant compared to the WT under hypoxia.

The presence of a zinc finger DNA binding domain in Pas2 suggests that it may function as a transcription factor. To dissect its gene regulatory role in response to hypoxia, we compared the transcriptomic profiles of *pas2*Δ cells with those of the wild-type strain under hypoxia. In total, 233 genes were differentially expressed, with 174 downregulated and 59 upregulated (Fig. 3A). GO analysis of these DEGs from the *pas2*Δ/WT comparison under hypoxia gave functional categories (Fig. 3B) similar to those derived from WT normoxia/WT hypoxia, as shown in Fig. 1A. The genes within these functional categories failed to be induced under hypoxia when *PAS2* was absent (Fig. 3B). As described above, hypoxia triggers global transcriptomic profile changes in wild-type C. neoformans. In particular, the transcript levels of metabolic genes encoding the enzymes that reprogram the energy production and redox balance were upregulated under hypoxia (Fig. 1C). Strikingly, the deletion of *PAS2* abolished the induction of almost all these hypoxia-responsive genes (Fig. 3C and D and Data Set S4), strongly suggesting the role of Pas2 in regulating hypoxia-induced metabolic reprogramming. For instance, the genes involved in glycolysis (*CNAG_05387* and *CNAG_06290*), fermentation (*CNAG_01955*, *CNAG_04659*, and *CNAG_07745*), gluconeogenesis (*CNAG_04217*), the PPP (*CNAG_04025* and *CNAG_06923*), and the TCA cycle (*CNAG_06374*) were highly induced in response to hypoxia in the wild type but not the *pas2*Δ mutant (Fig. 3C and Data Set S4). In addition, the induction of the flavohemoglobin-encoding gene *CNAG_01464* was abolished in the *pas2*Δ mutant in response to hypoxia. Together, these results strongly support that Pas2 regulates metabolic genes at the transcript level in response to hypoxia in C. neoformans. To our knowledge, Pas2 is the first identified transcription factor in C. neoformans that mediates such an extensive metabolic reprogramming in response to hypoxia.

10.1128/mBio.03602-20.8 DATA SET S4 List of DEGs in the *pas2*Δ mutant versus the wild type under hypoxia. Download Data Set S4, XLSX file, 0.02 MB. Copyright © 2021 Zhao and Lin. 2021 Zhao and Lin. https://creativecommons.org/licenses/by/4.0/ This content is distributed under the terms of the Creative Commons Attribution 4.0 International license.

To explore how individual metabolic pathways contribute to hypoxic adaptation mediated by Pas2, we took advantage of the partial genome deletion library made in the H99 background and examined the hypoxic growth of the mutants disrupted in the identified DEGs. Almost all of the single-gene deletion mutants tested grew like the wild-type strain under hypoxia conditions (Fig. S2). This finding is not unexpected given that a robust metabolism reprogramming likely involves multiple overlapping pathways to collectively facilitate hypoxic adaptation. Only global regulators of metabolic reprogramming will likely have a dramatic effect on hypoxia growth.

10.1128/mBio.03602-20.2 FIG S2 Hypoxic growth of the mutants disrupted in the identified DEGs through transcriptome analysis (Fig. 3). Cultures grown overnight were diluted to an OD_600_ of 3, serially diluted, spotted onto YPD medium, and cultured under normoxia and hypoxia for 2 days. Download FIG S2, TIF file, 2.0 MB. Copyright © 2021 Zhao and Lin. 2021 Zhao and Lin. https://creativecommons.org/licenses/by/4.0/ This content is distributed under the terms of the Creative Commons Attribution 4.0 International license.

### The PAS and zinc finger domains are required for the proper function of Pas2.

**FIG 4:**
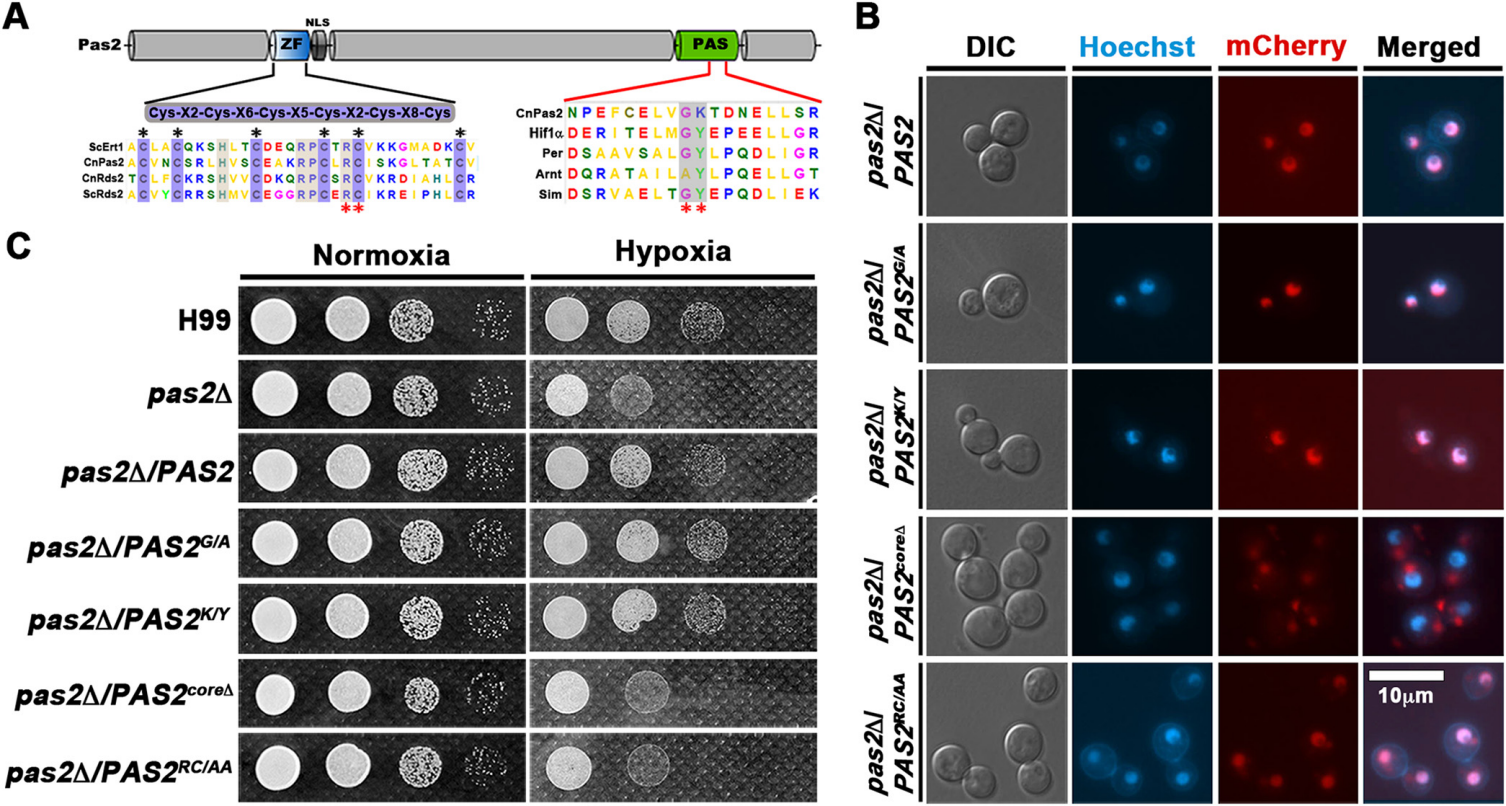
Both the PAS and zinc finger domains are required for the function of Pas2 in regulating hypoxia growth in C. neoformans. (A) Diagram of the Pas2 protein and the conserved sequence alignment of its PAS and zinc finger (ZF) domains. (B) Fluorescence of mCherry-tagged alleles of Pas2. Cells were cultured in liquid YPD medium at 30°C under ambient air overnight. Hoechst staining was used to visualize nuclei. DIC, differential interference contrast. (C) Assay of hypoxic growth of the *pas2*Δ mutant transformed with different alleles of *PAS2*.

Pas2 has one predicted PAS domain, a nuclear localization sequence (NLS), and a zinc finger domain (Fig. 4A). As expected, mCherry-tagged Pas2 localized to the nucleus (Fig. 4B), and it successfully restored the hypoxia growth defect when introduced into the *pas2*Δ mutant (Fig. 4C), indicating its functionality. In contrast to HIFs that have two tandem PAS domains (34), Pas2 has only one predicted PAS domain. Interestingly, the core amino acid K527 in the PAS domain of Pas2 differs from the amino acid Y present in other well-characterized PAS proteins in higher eukaryotes (Fig. 4A). To examine if this difference affects the function of Pas2, we conducted site-directed mutagenesis and obtained a mutated allele of Pas2^K/Y^ (Fig. 4A). mCherry-tagged Pas2^K/Y^ still localized to the nucleus like the wild-type allele (Fig. 4B). The overexpression of Pas2^K/Y^ with the constitutively active promoter P*_GPD1_* complemented the hypoxic growth defect in the *pas2*Δ mutant, confirming that Pas2^K/Y^ is functional (Fig. 4C). Likewise, a G526-to-A mutation did not affect the nuclear localization or the function of Pas2 in hypoxia growth (Fig. 4B and C). These results indicate that the polymorphisms in these conserved amino acids of the PAS domain do not alter the functionality of Pas2.

To examine whether the PAS domain itself is critical for Pas2’s regulation of hypoxic growth in C. neoformans, we deleted the core amino acids of the PAS domain in frame (Pas2^coreΔ^). Surprisingly, the fluorescence signal of the mCherry-tagged Pas2^coreΔ^ protein was enriched in vacuoles instead of the nucleus (Fig. 4B). Consistent with its mislocalization, Pas2^coreΔ^ failed to complement the hypoxic growth defect when introduced into the *pas2*Δ mutant (Fig. 4C). This result suggests that the intact PAS domain is required either for Pas2’s proper nuclear localization or for its stability.

The Zn_2_C_6_-type zinc finger DNA binding domain present in Pas2 also differs from the basic helix-loop-helix (bHLH) DNA binding motif present in HIFs. To test if the zinc finger binding domain is critical for the function of Pas2, we conducted site-directed mutation on the fifth conserved cysteine residue known to be critical for zinc binding activity and DNA binding activity (38) (Fig. 4A). The Pas2^C/A^ protein was still localized to the nucleus, but Pas2^C/A^ failed to complement the hypoxic growth defect in the *pas2*Δ mutant (Fig. 4B and C). Thus, the zinc finger DNA binding domain is critical for Pas2’s function in regulating hypoxia growth.

### Pas2 interacts with Rds2 in regulating hypoxic adaptation in C. neoformans.

**FIG 5:**
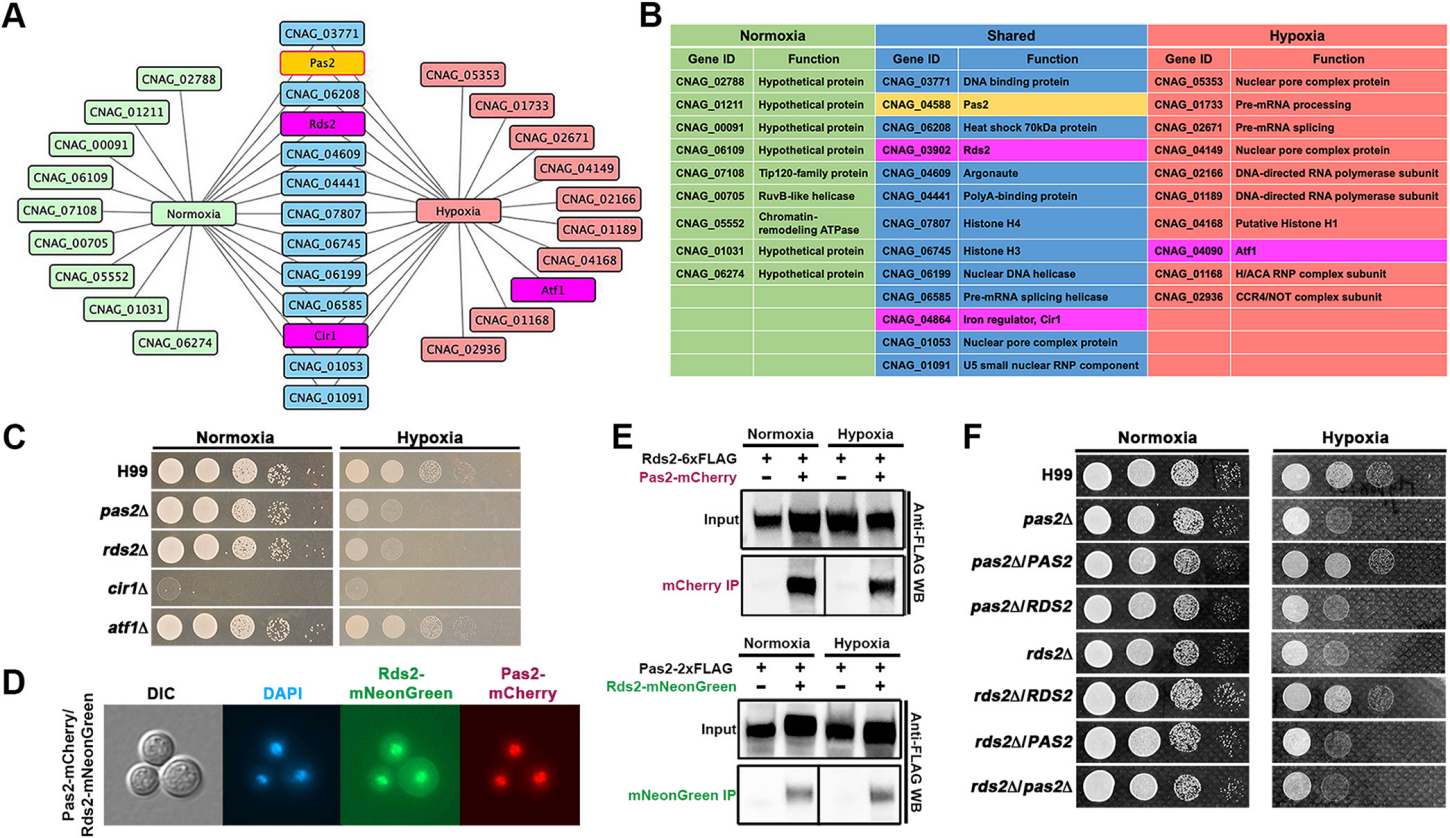
Pas2 interacts with Rds2 in regulating hypoxic adaptation in C. neoformans. (A) CoIP/MS hits of potential interacting partners of Pas2 under normoxia and hypoxia. Pas2 itself is highlighted in yellow, and three transcription factors are highlighted in purple. (B) Information on proteins from the CoIP/MS hits. (C) Hypoxic growth of three transcription factor mutants identified by CoIP/MS. (D) Fluorescence colocalization of Pas2 and Rds2. Rds2 and Pas2 were tagged with mNeonGreen and mCherry, respectively. Cells were cultured in liquid YPD medium at 30°C under ambient air overnight. DAPI staining was used to visualize nuclei. (E) Validation of the interaction between Pas2 and Rds2 by CoIP/WB. A Pas2-mCherry- and Rds2-6×FLAG-coexpressing strain was cultured under both normoxia and hypoxia conditions. Cells were fixed and lyophilized for total protein extraction. RFP-trap was used for pulldown, and anti-FLAG antibody was used in the subsequent WB assay. An Rds2-mNeonGreen- and Pas2-2×FLAG-coexpressing strain was used for the reciprocal CoIP/WB assay, in which mNeonGreen-trap was used for pulldown and anti-FLAG antibody was used for the subsequent WB assay. The strains expressing only FLAG-tagged Rds2 or Pas2 were included as negative controls. (F) Epistasis assay between *PAS2* and *RDS2*.

Pas2 is always localized in the nucleus under all the conditions tested (normoxia, hypoxia, and cobalt chloride conditions) (Fig. S3). To determine if Pas2 works with a partner in regulating hypoxic growth in C. neoformans, we conducted a coimmunoprecipitation assay coupled with mass spectrometry (CoIP/MS) with mCherry-tagged Pas2 to identify its interacting partners under normoxia and hypoxia. Because Pas2 is always localized to the nucleus, we focused on the candidates that could potentially localize to the nucleus. Out of the 22 potential nuclear proteins pulled down by Pas2, 9 were normoxia specific and 10 were hypoxia specific, with 13 shared ones (Fig. 5A and B and Data Set S6). Functional enrichment analyses of the potential nuclear partners strongly suggested that Pas2 may be involved in gene transcription, transcript processing, and transcript exporting (Fig. 5B). We were particularly interested in potential transcription factors as they may regulate gene transcription together with Pas2 as a heterocomplex. Three of the 22 potential candidates are annotated as transcription factors: Cir1, Afl1, and a protein encoded by *CNAG_03902*. Cir1 is the known regulator of iron homeostasis in C. neoformans (39). Afl1 plays important roles in the response to environmental stress and antifungal drug treatment. Afl1 is also important for the production of melanin and capsule in C. neoformans (40, 41). *CNAG_03902* encodes a zinc finger transcription factor that has not been studied in C. neoformans. It is homologous to *Saccharomyces RDS2* (regulator of drug sensitivity), which is involved in the regulation of gluconeogenesis (26). However, the role of Rds2 in regulating hypoxia adaptation in this facultative anaerobic model budding yeast has never been investigated based on the published literature.

10.1128/mBio.03602-20.3 FIG S3 Pas2 localizes to the nucleus under normoxia, hypoxia, and CoCl_2_ conditions (Fig. 4 and 5). Download FIG S3, TIF file, 1.3 MB. Copyright © 2021 Zhao and Lin. 2021 Zhao and Lin. https://creativecommons.org/licenses/by/4.0/ This content is distributed under the terms of the Creative Commons Attribution 4.0 International license.

10.1128/mBio.03602-20.9 DATA SET S5 List of Pas2-dependent genes in response to hypoxia and their GO categories. Download Data Set S5, XLSX file, 0.01 MB. Copyright © 2021 Zhao and Lin. 2021 Zhao and Lin. https://creativecommons.org/licenses/by/4.0/ This content is distributed under the terms of the Creative Commons Attribution 4.0 International license.

10.1128/mBio.03602-20.10 DATA SET S6 Potential Pas2-interacting proteins under hypoxia and normoxia. Download Data Set S6, XLSX file, 0.01 MB. Copyright © 2021 Zhao and Lin. 2021 Zhao and Lin. https://creativecommons.org/licenses/by/4.0/ This content is distributed under the terms of the Creative Commons Attribution 4.0 International license.

To determine if any of these three potential transcription factors are involved in hypoxic growth in C. neoformans, we tested the growth of *cir1*Δ, *afl1*Δ, and *rds2*Δ mutants under normoxia and hypoxia conditions. As shown in Fig. 5C, the deletion of *CIR1* resulted in a growth defect at 37°C, as reported previously (39). The deletion of *AFL1* had no effect on hypoxic growth (Fig. 5C). Interestingly, the deletion of *RDS2* impaired cryptococcal growth under hypoxia, recapitulating the phenotype caused by the deletion of *PAS2* (Fig. 5C). The introduction of the wild-type copy of *RDS2* driven by its own promoter complemented the hypoxic growth defect of the *rds2*Δ mutant (Fig. 5F), further confirming the role of Rds2 in hypoxia growth. This result indicates that Rds2 may interact with Pas2 and regulate cryptococcal adaptation to hypoxia.

As predicted, both Rds2 and Pas2 were localized in the nucleus based on the subcellular localization of the fluorescently tagged proteins (Fig. 5D). To validate the interaction between Pas2 and Rds2, we performed a reciprocal CoIP assay coupled with Western blotting (CoIP/WB) using cryptococcal strains with differently tagged Pas2 and Rds2 proteins. We found that mCherry-tagged Pas2 was able to pull down FLAG-tagged Rds2, and conversely, mNeonGreen-tagged Rds2 was able to pull down FLAG-tagged Pas2 (Fig. 5E), corroborating the physical interaction between these two transcription factors. The results also showed that Pas2 and Rds2 interact with each other under both normoxia and hypoxia conditions (Fig. 5E).

If Pas2 and Rds2 function as a heterocomplex similar to the Hif1α-Hif1β complex, then the overproduction of one factor would not be able to compensate for the loss of the other. Indeed, neither the overexpression of *PAS2* in the *rds2*Δ mutant nor the reciprocal overexpression of *RDS2* in the *pas2*Δ mutant could restore their hypoxic growth defect (Fig. 5F). In addition, the double deletion of *PAS2* and *RDS2* resembled the phenotypes of the single deletion of either *PAS2* or *RDS2* (Fig. 5F), indicating that Pas2 and Rds2 function in the same genetic pathway, in contrast to Pas2 and Stp1 (Fig. 2C). Thus, the genetic data demonstrate that both transcription factors are required for the optimal growth of cryptococcal cells under hypoxia. Together with our CoIP data, these results strongly suggest that Pas2 and Rds2 interact with each other in regulating hypoxic adaptation in C. neoformans.

### Regulation of the Pas2/Rds2 complex in hypoxia adaptation is Snf1 independent in C. neoformans.

**FIG 6:**
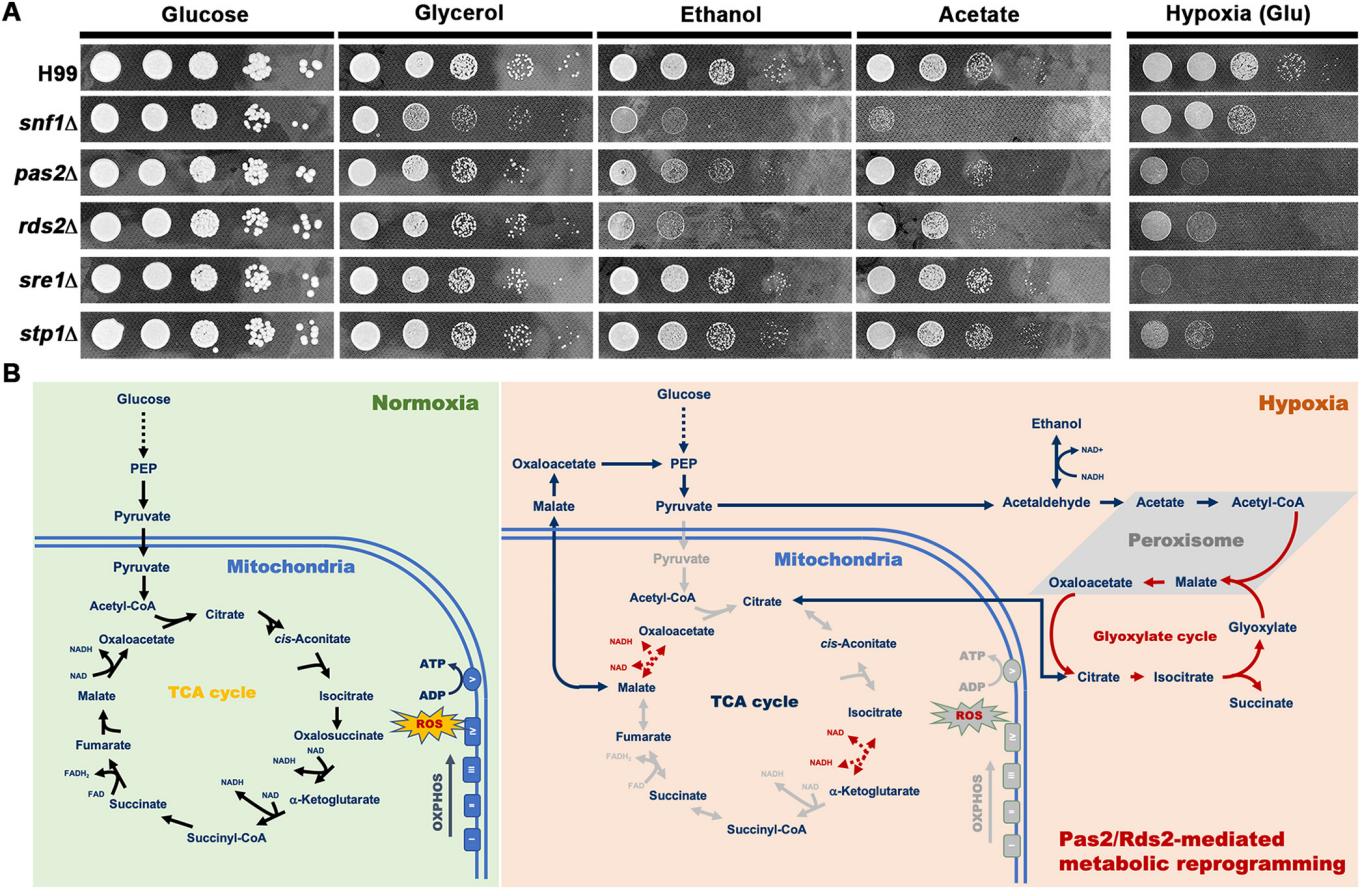
Pas2/Rds2 mediates metabolic reprogramming under hypoxia in C. neoformans. (A) Pas2 and Rds2 regulate hypoxic growth in addition to alternative carbon source utilization. The medium with 1% yeast extract and 2% peptone was used as the base medium. Two percent glucose, glycerol, ethanol, or sodium acetate was supplemented into the base medium. To test carbon source utilization, a spotting assay with serial dilutions was conducted under normoxia conditions. Hypoxic growth of the wild-type strain and mutants was tested on YPD medium. (B) Proposed model of Pas2/Rds2-mediated metabolic reprogramming under hypoxia in C. neoformans. Under normoxia, TCA cycle-coupled OXOPHOS is the main energy source to fuel the biological processes in aerobic cells. Under hypoxia, cells reprogram metabolism to avoid the accumulation of reactive oxygen species (ROS) due to the lack of oxygen as the final electron acceptor. The energy supply changes from oxygen-dependent OXOPHOS to glycolytic fermentation. Meanwhile, cells reshuffle the TCA cycle to avoid producing excessive reducing agents and subsequently prevent the accumulation of harmful ROS. Alternative metabolic pathways, including glyoxylate shunt and gluconeogenesis pathways, are upregulated to balance the redox homeostasis and compensate for the lack of building blocks. In addition, cells may reverse the TCA cycle into a reductive direction to maintain the carbon supply for acetyl-CoA, citrate, and fatty acids from glutamine in hypoxia. PEP, phosphoenolpyruvate; FAD, flavin adenine dinucleotide; FADH_2_, reduced flavin adenine dinucleotide.

In *Saccharomyces*, Rds2 is involved in Snf1-mediated utilization of alternative carbon sources (26). To test if Pas2 and Rds2 regulate the utilization of alternative carbon sources in *Cryptococcus*, we cultured wild-type H99, the *pas2*Δ mutant, and the *rds2*Δ mutant on media containing different carbon sources. The *Cryptococcus snf1*Δ mutant was included as a control. Consistent with the observation in budding yeast, the deletion of *SNF1* in C. neoformans severely impaired its utilization of ethanol, glycerol, or lactate as the sole carbon source (Fig. 6A). In comparison, the deletion of *PAS2* or *RDS2* only modestly reduced cryptococcal growth on the media with these alternative carbon sources (Fig. 6A). In stark contrast to the phenotypes on media containing various C sources, the deletion of *SNF1* had a much subtler effect on hypoxic growth in C. neoformans than the deletion of *RDS2* or *PAS2* (Fig. 6A), indicating that Pas2 and Rds2 function independently of Snf1 in response to hypoxia. Collectively, these results suggest that Rds2 and Pas2 regulate hypoxic adaptation in addition to their role in regulating alternative carbon source utilization in C. neoformans.

## DISCUSSION

For aerobic organisms, TCA cycle-coupled OXOPHOS provides the main energy source fueling biological processes under normoxia (Fig. 6B). Under hypoxia, these cells have to reprogram metabolism from oxygen-dependent OXOPHOS to glycolytic fermentation to supply energy. Meanwhile, cells also need to reshuffle the TCA cycle to avoid producing excessive reducing agents and prevent the accumulation of harmful reactive oxygen species (ROS). Alternative metabolic pathways, such as the glyoxylate shunt and gluconeogenesis pathways, are upregulated to balance redox homeostasis and to supply the building blocks (Fig. 6B).

Our comparative transcriptomic analyses under normoxia and hypoxia indicate an obvious metabolic reshuffle in C. neoformans from OXOPHOS to fermentation under hypoxia. Genes involved in glycolysis and fermentation were highly induced, while some mitochondrial genes related to OXOPHOS were downregulated (Fig. 1; see also Data Set S1 in the supplemental material). The metabolic pathways that contribute to redox balance, such as the glyoxylate shunt and GABA biosynthesis, were also upregulated (Fig. 1). The seemingly paradoxical upregulation of some genes involved in the TCA cycle might indicate that *Cryptococcus* reverses the TCA cycle to keep the supply of building blocks for other biosynthetic pathways and to prevent the accumulation of excessive NADH and consequent ROS with the suppression of OXPHOS under hypoxia (Fig. 6B). This hypothesis is consistent with previous observations in other fungi (11, 12, 42). In hypoxic tumor cells, *IDH1* and *IDH2* mediate the reversal of the TCA cycle (43). Consistently, key genes involved in the reductive branch of the TCA cycle, including the *IDH1* (*CNAG_07851*) and *IDH2* (*CNAG_07363*) homologs, were upregulated under hypoxia in *Cryptococcus* (Fig. 1C and Data Set S2). In addition, genes involved in the PPP (*CNAG_01984*, *CNAG_04025*, and *CNAG_06923*/*XFP2*) were also upregulated (Fig. 1), which likely help provide building blocks for biosynthesis and generate NADPH for the reduction of glutathione to defend against the accumulation of ROS. *XFP2* is involved in acetate biosynthesis in C. neoformans and other fungi (44, 45), during which ATP is generated without the consumption of oxygen. Given that acetate is one of the main metabolites in the hypoxic cryptococcoma *in vivo* (46–48), acetate production may contribute to hypoxia adaptation in C. neoformans (49). Collectively, the changes in the transcription of metabolic genes in response to hypoxia observed in C. neoformans here and in other organisms reported previously strongly support a conserved metabolic adaptation to hypoxia across lower and higher eukaryotes (Fig. 6B). Further metabolomic profiling studies would guide future investigation of the roles of these key metabolic pathways in hypoxia adaptation.

In higher eukaryotes like mammals, adaptation to hypoxia through metabolic reprogramming is controlled mainly by the HIFs. To our knowledge, there are no HIF homologs identified in lower eukaryotes. Here, we identified the transcription factor Pas2 that controls the transcript levels of metabolic genes, especially the glycolic and fermentation genes, leading to hypoxia adaptation in C. neoformans. Pas2 localizes to the nucleus under both normoxia and hypoxia conditions. We hypothesize that a partner interacting with Pas2 through the PAS domain may contribute to Pas2’s function in the nucleus (34). Indeed, we identified another transcription factor, Rds2, that interacts with Pas2 in regulating hypoxia adaptation (Fig. 6B). The homologs of Pas2 and Rds2 in S. cerevisiae and A. nidulans are known to regulate Snf1-mediated alternative carbon source utilization and gluconeogenesis (26, 27, 29). We found that Pas2 and Rds2 may regulate alternative carbon source utilization as part of the Snf1 regulon, but they mediate hypoxic adaptation in an Snf1-independent manner in C. neoformans. In addition to the genetic regulation executed by transcription factors, it has been shown that Snf5, as a component of the SWI/SNF chromatin remodeling complex, regulates hypoxic metabolic flexibility in C. albicans (50). It would be of great interest to investigate the cross talk between genetic and epigenetic regulation in response to hypoxia in fungal pathogens. Future studies on the impact of Pas2/Rds2-mediated metabolic rewiring on virulence in an animal model would provide novel insights into understanding cryptococcal pathogenesis.

## MATERIALS AND METHODS

### Strains.

The C. neoformans strains used in this study are listed in Table S1 in the supplemental material. Cryptococcal cells were maintained on YPD medium unless specified otherwise. Transformants obtained by biolistic transformation (51) or by transient CRISPR-Cas9 coupled with electroporation (TRACE) (52) were selected on YPD medium with 100 μg/ml of nourseothricin (NAT), 100 μg/ml of neomycin (NEO), or 200 μg/ml of hygromycin (HYG). For CoCl_2_ sensitivity assays, YES medium plus 0.6 mM CoCl_2_ was used. YES medium contains 0.5% (wt/vol) yeast extract, 3% glucose, and supplements consisting of 225 mg/ml each of uracil, adenine, leucine, histidine, and lysine (53). Strains to be tested were grown overnight in liquid YPD medium at 30°C with shaking. The cells were washed with sterile water, adjusted to the same cell density (optical density at 600 nm [OD_600_] of 3.0), and serially diluted. To analyze the growth on YES medium plus CoCl_2_, cell suspensions with the dilutions were spotted onto YES agar medium containing 0.6 mM CoCl_2_ and incubated at 30°C for 2 days. The hypoxic environment was maintained using a Biospherix C chamber with O_2_ levels controlled by a Pro-Ox controller and CO_2_ levels controlled by a Pro-CO_2_ controller (Biospherix, Lacona, NY, USA). To test the ability of the strains to grow under hypoxia, serial dilutions of cells were spotted onto YPD agar medium and grown in the hypoxic chamber with 0.1% oxygen and 5% CO_2_ at 37°C for 2 days.

10.1128/mBio.03602-20.4 TABLE S1 Key resources. Download Table S1, DOCX file, 0.02 MB. Copyright © 2021 Zhao and Lin. 2021 Zhao and Lin. https://creativecommons.org/licenses/by/4.0/ This content is distributed under the terms of the Creative Commons Attribution 4.0 International license.

### Gene manipulation.

To delete the *PAS2* open reading frame (ORF) in the *rds2*Δ and *stp1*Δ backgrounds, a deletion construct that contains approximately 1 kb of flanking sequences and the split dominant marker *NEO* and the constructs of *CAS9* and guide RNAs (gRNAs) were introduced into the *rds2*Δ and *stp1*Δ recipient strains by electroporation as described previously (52). The transformants generated were screened by two rounds of diagnostic PCRs. The first round of PCR was performed to detect the integration of the construct into the correct locus. The second round of PCR was performed to confirm the loss of the *PAS2* ORF. All primers used to make the gene deletion mutants are listed in Table S1.

For gene complementation, the ORFs plus approximately 1.0 kb of their upstream regions were amplified by PCR and cloned into vectors for tagging with mCherry, mNeonGreen, or FLAG. *SRE1* with its promoter region was amplified with the primer pair Linlab4895/YZ and Linlab4898/YZ, digested with NotI and FseI, and cloned into the digested plasmid pYZ75 to generate the FLAG-tagged plasmid pYZ105. *PAS2* with its promoter region was amplified with the primer pair Linlab4894/YZ and Linlab4303/YZ, digested with NotI and FseI, and cloned into the digested plasmid pYZ75 to generate the FLAG-tagged plasmid pYZ101. *RDS2* with its promoter region was amplified with the primer pair Linlab5066/YZ and Linlab5770/YZ, digested with NotI, and cloned into SUN-pHYG-4×FLAG (54) to generate the FLAG-tagged plasmid pYZ67.

For gene overexpression with a constitutively active promoter, the constructs were created by amplifying the entire ORF by PCR and cloned into vectors behind the *GPD1* promoter or the *TEF1* promoter. The ORF of *PAS2* was amplified with the primer pair Linlab4302/YZ and Linlab4436/YZ, digested with AsiSI and FseI, and cloned to generate the P*_GPD1_*-driven and mCherry-tagged *PAS2* plasmid pYZ97. To overexpress *PAS2* with the promoter of *TEF1*, the ORF of *PAS2* was amplified with the primer pair Linlab4445/YZ and Linlab4403/YZ, digested with SmaI and FseI, and cloned into pYZ75 to generate the plasmid pYZ79. To overexpress *RDS2* with the promoter of *GPD1*, the ORF of *RDS2* was amplified with the primer pair Linlab5067/YZ and Linlab5068/YZ, digested with AsiSI, and cloned into pYZ194 to generate the plasmid pYZ190.

For site-directed mutagenesis and truncation, fusion PCR was used to introduce nucleotide mutations or truncations into the Pas2 protein-coding sequence. In brief, the ORF of *PAS2* was split into two parts with overhangs at the mutation loci and amplified by PCR using specifically designed primers to change the codons of conserved residues. Next, these two parts were fused through fusion PCR and cloned into AsiSI- and FseI-digested vector pYZ97 to swap the wild-type allele of *PAS2*. The tagged alleles were then introduced into the safe haven (55) of recipient strains by TRACE (52). Stable transformants were selected after stability testing with five passages on nonselective medium and further analyzed by diagnostic PCR to confirm the replacement as described previously (65). All primers and plasmids used in this study are listed in Table S1.

### Microscopic examination.

The mCherry- or mNeonGreen-tagged strains were cultured overnight in liquid YPD medium and observed under a Zeiss Imager M2 microscope. Images were acquired with an AxioCam MRm camera and processed with Zen pro software (Carl Zeiss Microscopy). Nuclei were visualized by live staining with Hoechst or DAPI (4′,6-diamidino-2-phenylindole) stain after formaldehyde fixation as described previously (56).

### RNA-seq and data analysis.

For transcriptome analysis in response to hypoxia, cultures grown overnight in liquid YPD medium were diluted to an OD_600_ of 0.03. Two hundred microliters of the diluted cells was plated onto YPD plates and cultured overnight at 30°C under normoxia. Half of the plates were then transferred into the hypoxia chamber (0.1% O_2_ and 5% CO_2_ at 37°C), and half of them were transferred to the control conditions (20 to 21% O_2_ and 5% CO_2_ at 37°C). The cells were quickly sampled and snap-frozen with liquid nitrogen after 3 h. The total RNA was extracted using a PureLink RNA minikit (Life Technologies) according to the instructions of the manufacturer. Direct poly(A) RNA sequencing was performed by the Texas A&M AgriLife Genomics and Bioinformatics Service. Preliminary quality analysis of the raw FASTQ files was conducted using FastQC (57), and trimming of low-quality bases was performed with a custom perl script. STAR (58) was used to map the processed reads to the reference genome. The DESeq2 program (59) was used to identify differentially expressed genes (DEGs) with the read counts from STAR as the input files. The functional analysis of DEGs was conducted using DAVID (60, 61), and the results were visualized by using the R packages ggplot2 (62) and GOplot (63).

### Protein extraction, Western blotting, and CoIP/MS.

Proteins were extracted from *Cryptococcus* strains according to a previously described method (64). Aliquots of proteins were separated on 4%-to-12% gradient SDS-PAGE gels (GenScript) and then transferred to a polyvinylidene difluoride (PVDF) membrane for Western blot analysis using the anti-FLAG antibody (Sigma). Protein aliquots from mNeonGreen-tagged or mCherry-tagged strains were processed for CoIP using mNeonGreen-trap (ChromoTek) or red fluorescent protein (RFP)-trap (ChromoTek) according to the manufacturer’s protocol. For CoIP/mass spectrometry (MS) assays, CoIP samples were processed and analyzed by the University of Georgia (UGA) proteome facility center for mass spectroscopy. The details about the antibodies, mNeonGreen-trap, and RFP-trap used in this study are provided in Table S1.

### Data availability.

The RNA-seq data have been deposited in the NCBI database under BioProject accession number PRJNA662775.
