# Supplementary figures and images for: A PAS Protein Directs Metabolic Reprogramming during Cryptococcal Adaptation to Hypoxia
Source: mBio. 2021 Mar 16;12(2):e03602-20. doi: 10.1128/mBio.03602-20 (PMC8092316; doi:10.1128/mBio.03602-20)

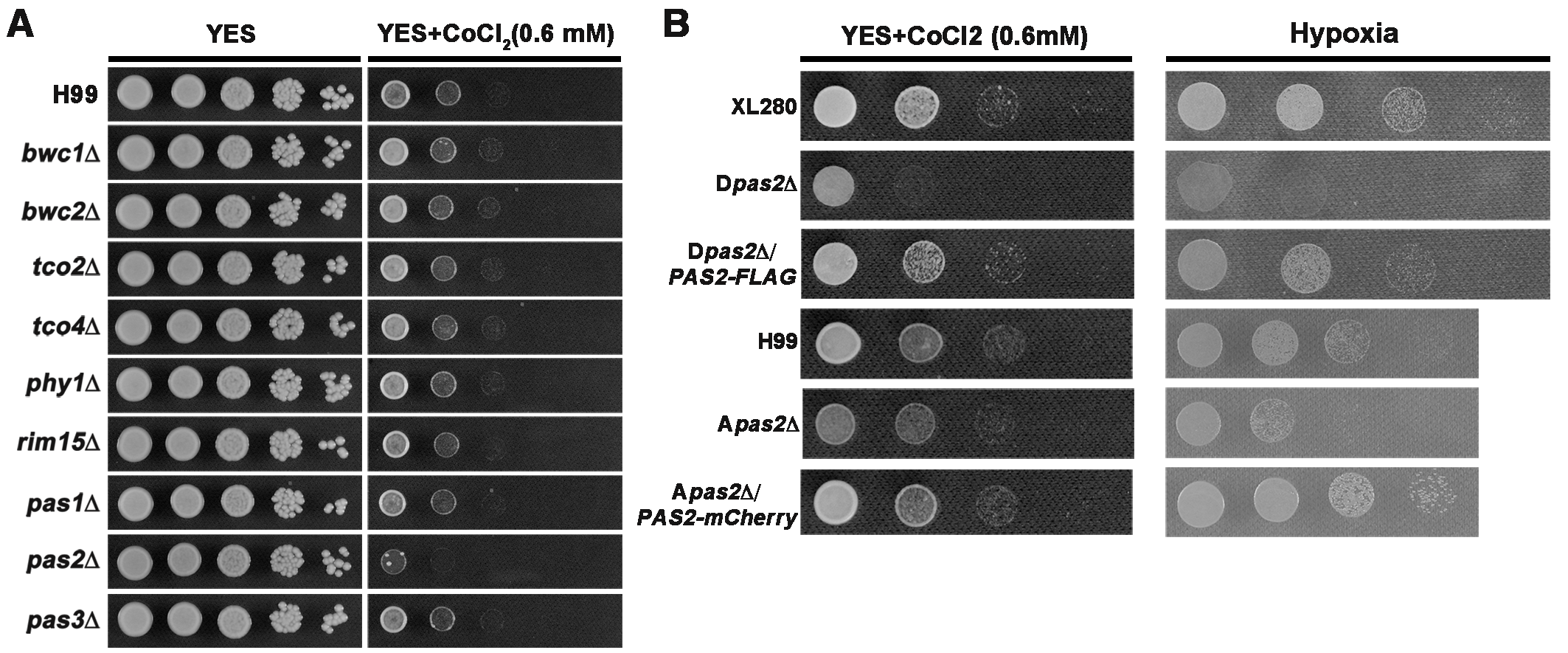

Supplement: FIG S1 [file mBio.03602-20-sf001.tif]

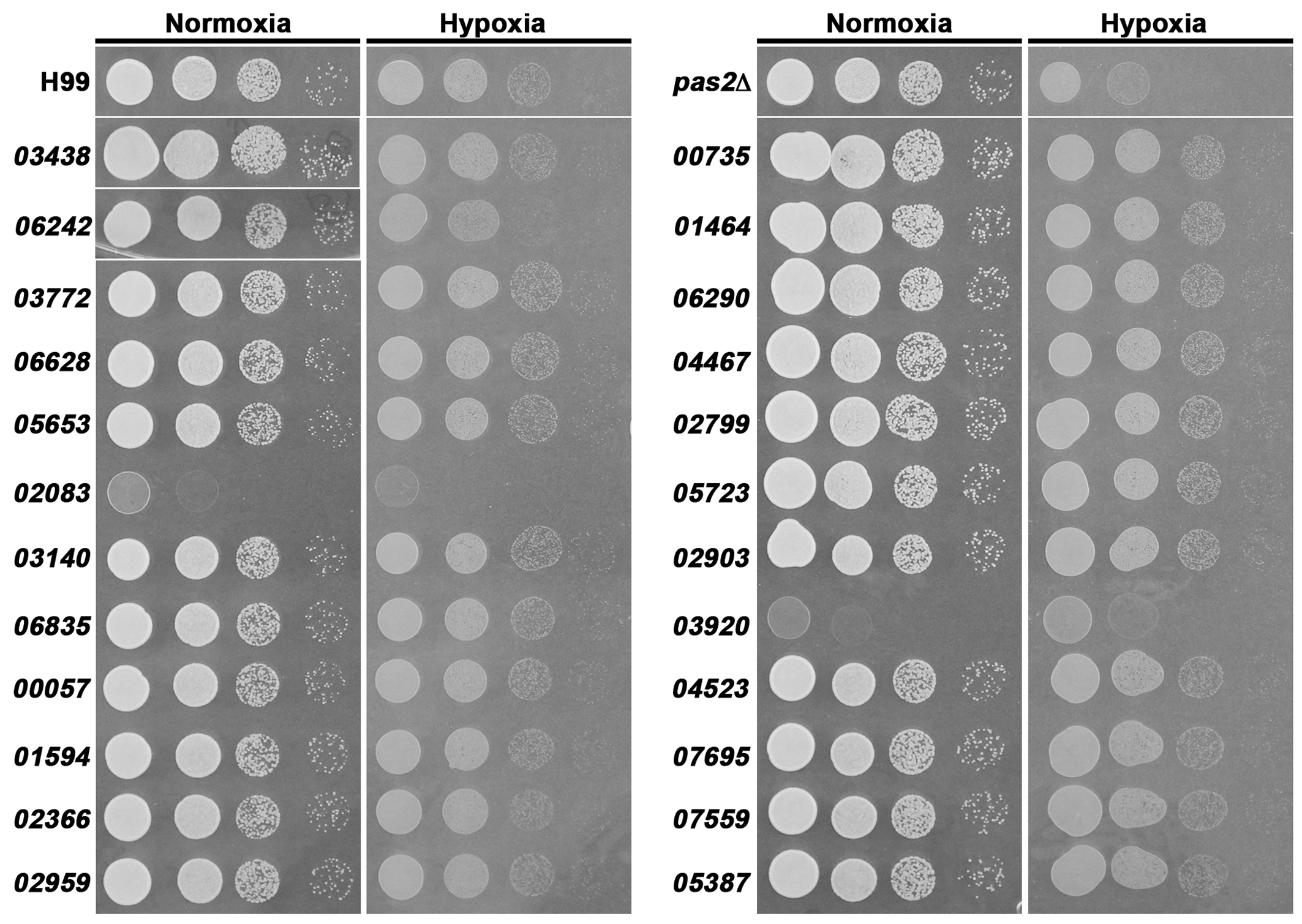

Supplement: FIG S2 [file mBio.03602-20-sf002.tif]

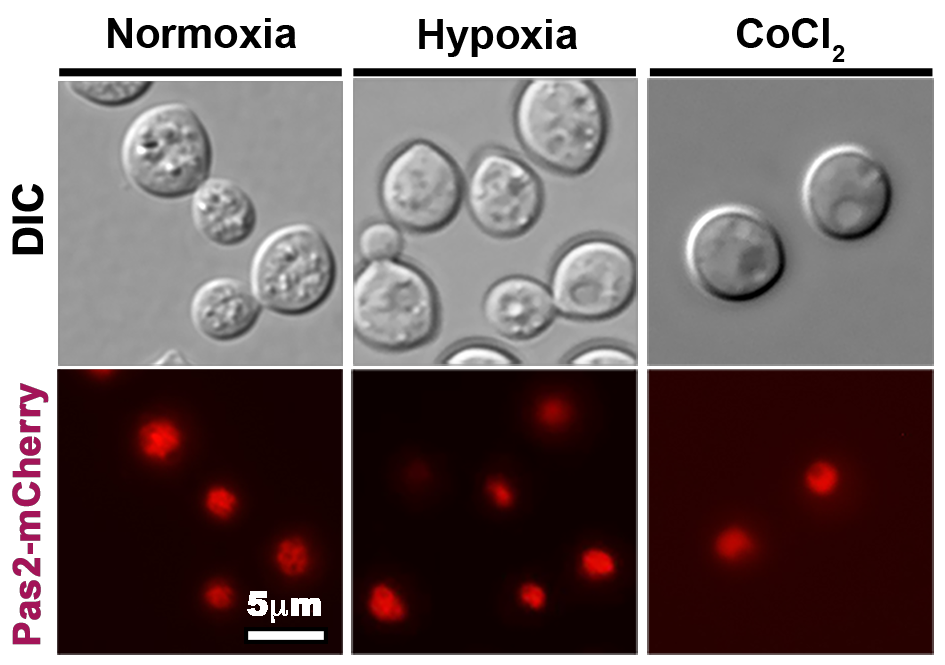

Supplement: FIG S3 [file mBio.03602-20-sf003.tif]
