# Supplementary material for: A PAS Protein Directs Metabolic Reprogramming during Cryptococcal Adaptation to Hypoxia
Source: mBio. 2021 Mar 16;12(2):e03602-20. doi: 10.1128/mBio.03602-20 (PMC8092316; doi:10.1128/mBio.03602-20)
Supplement: TABLE S1 [file mBio.03602-20-st001.docx]

| REAGENT or RESOURCE | SOURCE | IDENTIFIER |
| --- | --- | --- |
| Fungal Strains | | |
| XL280 | (49) | *C. neoformans* serotype D, wild type, MATalpha, |
| H99 | (50) | *C. neoformans* serotype A, wild type, MATalpha, |
| Linlab4756 | FGSC deletion set 2H5 | MATalpha, serotype A, *BWC1*::NAT |
| Linlab4757 | FGSC deletion set 1E7 | MATalpha, serotype A, *BWC2*::NAT |
| Linlab4760 | FGSC deletion set 0A7 | MATalpha, serotype A, *TCO2*::NAT |
| Linlab4761 | FGSC deletion set 9E1 | MATalpha, serotype A, *TCO4*::NAT |
| Linlab4758 | FGSC deletion set 0C11 | MATalpha, serotype A, *PHY1*::NAT |
| Linlab4759 | FGSC deletion set 9F9 | MATalpha, serotype A, *RIM15*::NAT |
| Linlab4762 | FGSC deletion set 0F5 | MATalpha, serotype A, *PAS1*::NAT |
| Linlab5759 | (52) | MATalpha, serotype A, *PAS2*::NAT |
| Linlab4764 | YZ3 | MATalpha, serotype A, *PAS3*::NAT |
| Linlab5285 | (52) | MATalpha, serotype A, *SRE1*::NAT |
| Linlab5289 | FGSC deletion set 0G11 | MATalpha, serotype A, *STP1*::NAT |
| Linlab5746 | (53) | MATalpha, serotype A, *SNF1*::NAT |
| Linlab5751 | (52) | MATalpha, serotype A, *RDS2*::NAT |
| Linlab6006 | This manuscript | MATalpha, serotype A, *STP1*::NAT, *PAS2*::NEO |
| Linlab5990 | This manuscript | MATalpha, serotype A, *RDS2*::NAT, *PAS2*::NEO |
| Linlab5454 | This manuscript | MATalpha, serotype A, *SRE1*::NAT, *P_SRE1_*-*SRE1*-2xFLAG-HYG |
| Linlab5455 | This manuscript | MATalpha, serotype A, *SRE1*::NAT, *P_PAS2_*-*PAS2*-2xFLAG-HYG |
| Linlab5443 | This manuscript | MATalpha, serotype A, *PAS2*::NAT, *P_SRE1_*-*SRE1*-2xFLAG-HYG |
| Linlab6100 | This manuscript | MATalpha, serotype A, *PAS2*::NAT, *P_RDS2_*-*RDS2*-mNeonGreen-HYG |
| Linlab6106 | This manuscript | MATalpha, serotype A, *RDS2*::NAT, *P_RDS2_*-*RDS2*-mNeonGreen-NEO |
| Linlab5886 | This manuscript | MATalpha, serotype A, *RDS2*::NAT, *P_GPD1_-PAS2*-mCherry-NEO |
| Linlab5435 | This manuscript | MATalpha, serotype A, *PAS2*::NAT, *P_GPD1_-PAS2*-mCherry-NEO |
| Linlab5850 | This manuscript | MATalpha, serotype A, *PAS2*::NAT, *P_GPD1_-PAS2^G/A^*-mCherry-NEO |
| Linlab5860 | This manuscript | MATalpha, serotype A, *PAS2*::NAT, *P_GPD1_-PAS2^K/Y^*-mCherry-NEO |
| Linlab5977 | This manuscript | MATalpha, serotype A, *PAS2*::NAT, *P_GPD1_-PAS2^core^*^Δ^-mCherry-NEO |
| Linlab6252 | This manuscript | MATalpha, serotype A, *PAS2*::NAT, *P_GPD1_-PAS2^RC/AA^*-mCherry-NEO |
| Linlab7502 | This manuscript | MATalpha, serotype A, *PAS2*::NAT, *P_GPD1_*-*PAS2*-mCherry-NEO, *P_RDS2_*-*RDS2*-mNeonGreen-HYG |
| Linlab6356 | This manuscript | MATalpha, serotype A, *PAS2*::NAT, *P*_GPD1_-*PAS2*-mCherry-NEO, *P_RDS2_*-*RDS2*-4xFLAG-HYG |
| Linlab6140 | This manuscript | MATalpha, serotype A, *P_TEF1_-PAS2*-2xFLAG-HYG, *P_GPD1_-RDS2*-mNeonGreen-NEO |
| Linlab4683 | This manuscript | MATalpha, serotype D, *PAS2*::NAT |
| Linlab5846 | This manuscript | MATalpha, serotype D, *PAS2*::NAT, *P_GPD1_-PAS2*-mCherry-NEO |
| Linlab7017 | (37) | MATalpha, serotype A, *CIR1*::NAT |
| Linlab7178 | FGSC deletion set 41G11 | MATalpha, serotype A, *ATF1*::NAT |
| Plasmids | | |
| pYZ97 | This manuscript | *P_GPD1_-PAS2*-mCherry-NEO |
| pYZ202 | This manuscript | *P_GPD1_-PAS2^G/A^*-mCherry-NEO |
| pYZ201 | This manuscript | *P_GPD1_-PAS2^K/Y^*-mCherry-NEO |
| pYZ95 | This manuscript | *P_GPD1_-PAS2^core^*^Δ^-mCherry-NEO |
| pYZ193 | This manuscript | *P_GPD1_-PAS2^RC/AA^*-mCherry-NEO |
| pYZ79 | This manuscript | *P_TEF1_-PAS2*-2xFLAG-HYG |
| pYZ190 | This manuscript | *P_GPD1_-RDS2*-mNeonGreen-NEO |
| pYZ67 | This manuscript | *P_RDS2_-RDS2*-4xFLAG-HYG |
| pYZ101 | This manuscript | *P_PAS2_-PAS2*-2xFLAG-HYG |
| pYZ57 | This manuscript | *P_RDS2_-RDS2*-mNeonGreen-NEO |
| pYZ124 | This manuscript | *P_RDS2_-RDS2*-mNeonGreen-HYG |
| pYZ105 | This manuscript | *P_SRE1_-SRE1*-2xFLAG-HYG |
| pYZ75 | This manuscript | *P_TEF1_-*2xFLAG-HYG |
| pYZ194 | This manuscript | *P_GPD1_*-mNeonGreen-NEO |
| Chemicals | | |
| Hygromycin | Research Products International Corp. | H75000 |
| G418 | Research Products International Corp. | G64000 |
| Nourseothricin | Jena Bioscience | AB-102-25G |
| CoCl_2_ | Millipore Sigma | 7646-79-9 |
| DAPI | ThermoFisher | D1306 |
| Hoechst | ThermoFisher | 62249 |
| Antibody and trap kit | | |
| Anti-FLAG | Millipore Sigma | F3165 |
| RFP-trap | ChromoTek | rtma-20 |
| mNeonGreen-trap | ChromoTek | ntma-20 |
| Experimental Models: Organisms/Strains | | |
| Software and Algorithms | | |
| R 3.6 | The R Foundation | https://www.rproject.org/ |
| ggplot2 | (54) | https://ggplot2.tidyverse.org/ |
| Oligonucleiotides | | |
| M13F | GTAAAACGACGGCCAG | Amplify drug marker |
| M13R | CAGGAAACAGCTATGAC | Amplify drug marker |
| Linlab3077/YZ | CGCGAGAAGACGATGTGA | Left forward primer for PAS2 deletion |
| Linlab3078/YZ | CTGGCCGTCGTTTTACTAGTTTGGCGGTTGCTG | Left reverse primer for PAS2 deletion |
| Linlab3079/YZ | GTCATAGCTGTTTCCTGAAACTGAATTGTTGAATGGA | Right forward primer for PAS2 deletion |
| Linlab3080/YZ | AGGCTTCTGAAGGGCTAG | Right reverse primer for PAS2 deletion |
| Linlab5110/YZ | TACGCGCTTGGTGATATACCAACAGTATACCCTGCCGGTG | Reverse primer to amplify gRNA for PAS2 deletion |
| Linlab5111/YZ | GGTATATCACCAAGCGCGTAGTTTTAGAGCTAGAAATAGCAAGTT | Forward primer to amplify gRNA for PAS2 deletion |
| Linlab4302/YZ | ATAGGCCGGCCATGCAAGACCACCAGTGG | PAS2-F-FseI |
| Linlab4303/YZ | ATAGGCCGGCCCTTGCGTCTCAGAGGGGATC | PAS2-R-FseI |
| Linlab4436/YZ | ATAGCGATCGCTTGCGTCTCAGAGGGGATC | PAS2-R-AsiSI |
| Linlab4894/YZ | AAGGAAAAAAGCGGCCGCTGGGTCCGTTGATTCTCC | P_PAS2_-F-NotI |
| Linlab4895/YZ | AAGGAAAAAAGCGGCCGCAAATCGGCTGATCTTGTG | P_SRE1_-F-NotI |
| Linlab4898/YZ | ATA GGCCGGCC C CAGTTCATCTAAATCGCCCT | SRE1-R-FseI |
| Linlab4445/YZ | ATACCCGGGATGCAAGACCACCAGTGG | PAS2-F-smaI |
| Linlab5066/YZ | ATAGCGGCCGCCAAGGGTATTCTGGCACG | P_RDS2_-F-NotI |
| Linlab5067/YZ | CCATTGCGATCGCTTGTATAGGCTAGTATCATTCA | RDS2-R-AsiSI |
| Linlab5068/YZ | ATAGCGATCGCATGCAGAACAACGCCCCCA | RDS2-F-AsiSI |
| Linlab5770/YZ | ATAGCGGCCGCTTGTATAGGCTAGTATCATTCA | RDS2-R-NotI |
| Linlab4922/YZ | GCACCTTGAAGCGCATGA | Primer for Pas2^RC/AA^ |
| Linlab5418/YZ | CCTTTTTAACAGCTGCCGTACACGG | Primer for Pas2^RC/AA^ |
| Linlab5419/YZ | CCGTGTACGGCAGCTGTTAAAAAGG | Primer for Pas2^RC/AA^ |
| PactinR | TTGTGCTCGGATGTGCGATAG | Primer for Pas2^RC/AA^ |
| Linlab5076/YZ | CTCACAAAACTCTGGATTGGCA | Primer for Pas2^coreD^ |
| Linlab5077/YZ | AATCCAGAGTTTTGTGAGGCCAAGCCTTCGTGAGTA | Primer for Pas2^coreD^ |
| Linlab4469/YZ | GAGTTTTGTGAGCTAGTGGCTAAGACGGATAATGAGTTGC | Primer for Pas2^G/A^ |
| Linlab4470/YZ | GCAACTCATTATCCGTCTTAGCCACTAGCTCACAAAACTC | Primer for Pas2^G/A^ |
| Linlab4471/YZ | GAGTTTTGTGAGCTAGTGGGTTATACGGATAATGAGTTGCTTTCG | Primer for Pas2^K/Y^ |
| Linlab4472/YZ | CTCAAAACACTCGATCACCCAATATGCCTATTACTCAACGAAAGC | Primer for Pas2^K/Y^ |
